# Morphological and Molecular Characterization of Apple Scab (*Venturia inaequalis*) in Kazakhstan and Kyrgyzstan

**DOI:** 10.3390/cimb47121011

**Published:** 2025-12-02

**Authors:** Valeriya Kostyukova, Alexandr Pozharskiy, Marina Khusnitdinova, Gulnaz Nizamdinova, Dilyara Gritsenko

**Affiliations:** 1 Laboratory of Molecular Biology, Institute of Plant Biology and Biotechnology, Almaty 050040, Kazakhstan; valera.kostykova.15@gmail.com (V.K.); aspozharsky@gmail.com (A.P.); germironame@gmail.com (M.K.); nizamdin13@gmail.com (G.N.); 2 Research Center AgriBioTech, Almaty 050040, Kazakhstan; 3 Faculty of Biology and Biotechnology, Al-Farabi Kazakh National University, Almaty 050040, Kazakhstan

**Keywords:** *Malus domestica*, *Malus sieversii*, morphological characterization, molecular identification, phylogenetic analysis

## Abstract

Apple scab, caused by the fungus *Venturia inaequalis*, is one of the most widespread and economically significant diseases of apple orchards, leading to reduced photosynthesis, fruit damage, and yield losses of up to 70%. In this study, a survey of 30 wild and cultivated apple populations in Kazakhstan and Kyrgyzstan was conducted, encompassing 302 samples. The pathogen was detected in 8 populations (48 samples), corresponding to an infection rate of 16%. Molecular identification using the *EF-1α* marker and ITS region sequencing definitively confirmed the presence of *V. inaequalis* in all positive samples. Phylogenetic analysis showed clear population structuring: isolates from Kyrgyzstan formed a distinct clade close to international lineages, while Kazakh isolates showed high genetic variation. These findings highlight the ongoing presence of *V. inaequalis* in Central Asia and emphasize the importance of combining morphological, molecular, and phylogenetic methods for effective pathogen monitoring and control.

## 1. Introduction

Apple scab, caused by the fungus *Venturia inaequalis*, is one of the most widespread and economically important diseases affecting apple orchards worldwide. The infection leads to significant yield losses: leaf damage reduces photosynthesis, disrupts gas exchange and transpiration, and causes premature tissue senescence. On fruits, the disease results in skin cracking, deformities, and reduced storage quality, thereby decreasing the market value of the produce [1]. Under conditions of severe disease development, skin damage also increases fruit susceptibility to secondary infections [2].

Crop losses can reach as high as 70% [3]. This often requires increased plant protection efforts, raising costs for fungicides and fertilizers [4]. Under unfavorable climatic conditions, especially high humidity and moderate temperatures, the disease can lead to extensive leaf drop and serious fruit damage, greatly lowering the commercial viability of the harvest [5].

The life cycle of *V. inaequalis* involves alternating sexual and asexual stages. The pathogen overwinters as pseudothecia on fallen leaves, where asci with ascospores are formed. After infecting young leaves and fruitlets, the fungus rapidly shifts to conidial sporulation; conidia enable multiple cycles of secondary infection throughout the season. This combination of resilient overwintering structures and a high rate of reinfection is a key factor in the epidemic nature of the disease and complicates its control [6,7].

The geographic distribution of *V. inaequalis* covers nearly all regions of the world. The pathogen has been reported in Europe, North America, Asia, as well as in certain parts of Africa and South America. Genetic studies suggest Central Asia as the likely center of origin for *V. inaequalis* [8]. Population diversity studies show that the eastern forests of wild apple (*Malus sieversii*) act as “relict” reservoirs of the fungus, from which its global spread probably started [8,9]. It is believed that, along with apple domestication, the pathogen migrated along trade routes, including the Silk Road, aiding its colonization of Europe and subsequent worldwide distribution [10].

*Malus sieversii*, native to Central Asia, is the main ancestor of the modern cultivated apple (*M. × domestica*). Genetic analyses confirm that current apple cultivars descend from this species, which naturally occurs in Kazakhstan, Kyrgyzstan, and nearby regions [11]. Populations of *V. inaequalis* found on wild *M. sieversii* in remote Kazakh forests display unique allele patterns, indicating the ancient origin of these fungal lineages [10]. Contemporary phytopathological studies in Kazakhstan verify the active presence of *V. inaequalis* in both cultivated orchards and wild populations of apple and pear [12,13,14].

This study presents data on the occurrence of *Venturia inaequalis* in Kazakhstan and Kyrgyzstan. The research includes surveys of previously unstudied apple populations, encompassing both wild forms and trees in abandoned orchards. This approach allows for characterization of the presence and prevalence of apple scab across different habitat types, including areas without regular maintenance. The results provide a foundation for further comprehensive analyses, including morphological and molecular studies of isolates, detailed mapping of infection foci, and assessment of the pathogen’s phylogenetic relationships and genetic variability at the local level. Such an approach facilitates a deeper understanding of the pathogen’s circulation in the region and its potential pathways of spread.

## 2. Materials and Methods

### 2.1. Collection and Morphology of Venturia inaequalis Isolates

Fruits of cultivated and wild apple trees displaying characteristic symptoms of scab—dark brown velvety spots on fruits and leaves—were collected in November 2024 from commercial and abandoned orchards in the Almaty and Zhetysu regions, as well as in the mountainous areas of Kyrgyzstan and Kazakhstan. A total of 30 populations were surveyed across lowland (400–600 m above sea level), foothill (700–1000 m above sea level), and mountainous regions (1000 m and above). Samples were transported as quickly as possible while maintaining a temperature of +4 °C.

The assessment of apple scab infection in the populations was carried out according to the method described in [15]. Infection severity was determined using a 9-point scale. Table 1 presents the values reflecting the degree of infection in the studied populations.

To obtain pure cultures of the pathogen, the method described by Xiancheng Li et al. [16] was used. A small fragment from the infected leaf area was excised and placed into a tube containing 2 mL of distilled water. After brief mixing, 200 µL of the suspension was applied to the surface of water agar and spread evenly. Plates were incubated at 18 °C for 24 h. Individual germinated conidia were selected under a light microscope (Evos M5000, Invitrogen, Thermo Fisher Scientific, Waltham, MA, USA) and transferred to potato dextrose agar (PDA). Cultures were incubated at 20 °C, and after 2–3 weeks, isolated colonies were transferred to fresh PDA plates, with regular monitoring of the pathogen’s growth characteristics. Isolation of cultures was performed in three replicates. The collection of isolates was stored at 4 °C.

For additional infection analysis, microscopic observations were conducted using light microscopy. Microscope slides were prepared by suspending fungal mycelium in water, followed by fixation through heating over an open flame. Staining was performed with a 0.025% aniline blue solution in lactoglycerol (a mixture of glycerol, lactose, and water in a 1:1:1 ratio), supplemented with 5% benzyl alcohol.

### 2.2. Real-Time PCR Identification

DNA was extracted from 21-day-old pure fungal cultures using the commercial Plant/Fungi DNA Isolation Kit (Norgen Biotek Corp., Thorold, ON, Canada) according to the manufacturer’s protocol, with prior mycelium homogenization in liquid nitrogen. Real-time PCR reactions were performed using Luna Universal Probe qPCR Master Mix (New England Biolabs, Ipswich, MA, USA) following the manufacturer’s instructions, with amplification carried out using specifically designed primers (Table 2). The analysis was ran on the CFX Opus 96 System, (BioRad, Hercules, SA, USA). The number of technical replicates was three, and deionized water was used as a negative control. The Cq values were calculated automatically using the threshold set by the instrument software(CFX Opus Maestro v.2.3).

### 2.3. Targeted DNA Sequencing Using the Oxford Nanopore Technologies (ONT) Platform

A representative fragment of the ITS region was amplified using the primers listed in Table 1. The PCR reaction mixture (25 µL) contained 1 U of Taq DNA polymerase (New England Biolabs), 1× Taq buffer, 0.2 mM of dNTPs, 0.2 µM of each primer, and 20 ng of template DNA. The amplification program began with an initial denaturation at 94 °C for 3 min, followed by 30 cycles of 94 °C for 30 s, 55 °C for 30 s, and 72 °C for 1 min, ending with a final elongation at 72 °C for 10 min. Amplicons of the expected length (466 bp) were verified on a 1.5% agarose gel, and DNA concentration was measured using a Qubit Flex fluorometer (Invitrogen) prior to library preparation for sequencing.

Sequencing was performed on a MinION Mk1B platform (Oxford Nanopore Technologies, Oxford, UK) using the SQK-RBK114.96 library preparation kit, following the manufacturer’s protocol. Reads were generated in high-accuracy mode with standard quality filtering. Basecalling and barcode trimming were performed using Dorado v7.4.12. Sequence alignment against the reference sequence (OQ110533.1) was conducted using minimap2.

### 2.4. Data Analysis

R (v. 4.5.0) with the ggplot package was used for visualization and analysis of the phylogenetic data. For phylogenetic analysis, additional sequences were retrieved from the NCBI database. Sequence alignment was performed using the MAFFT algorithm [19] in UGENE v.50.0 [20]. The phylogenetic tree was built using the UPGMA method based on the Maximum Composite Likelihood model, with branch support evaluated through 1000 bootstrap replicates in MEGA 11 [21]. Further editing and visualization of the tree were completed in FigTree v.1.4.4.

Standard tools were used for the statistical analysis of real-time PCR data, with calculations performed in accordance with previously published methods [22].

### 2.5. Mapping the Circulation of Fungal Pathogens

Geospatial analysis of *Venturia* fungal pathogen circulation in wild and cultivated apple (*Malus* spp.) populations was conducted using the QGIS 3.28.12—Firenze software environment. To build the geoinformation database, current satellite data in the WGS 84 coordinate system (EPSG:4326) were used, ensuring precise georeferencing of sampling locations. Coordinates for the 30 sampling sites were collected with a Garmin Montana 750i GPS receiver (Garmin, Olathe, KS, USA).

## 3. Results and Discussion

### 3.1. Morphological Analysis

A total of 30 populations of wild and cultivated apple trees in Kazakhstan and the Kyrgyz Republic were surveyed, including 302 samples. The apple scab pathogen was found in 8 populations (48 samples), resulting in an overall infection rate of 16%. Detailed data on the populations is available in Table 3.

The severity of infection varied among populations. In actively cultivated orchards in the Almaty region, individual trees were mildly affected: on the scale [15], values ranged from 2 to 3. In percentage terms for the entire population, this amounted to 30–32%. These results reflect regular fungicide application, although effectiveness may be reduced due to the development of evolutionarily driven resistance. In abandoned orchards in the Almaty region, infection was more pronounced, ranging from 60–73%, with individual trees showing values of 4–6, indicating a high level of scab spread. In wild populations, nearly complete leaf infection was observed, with values of 4–7.

**Figure 1:**
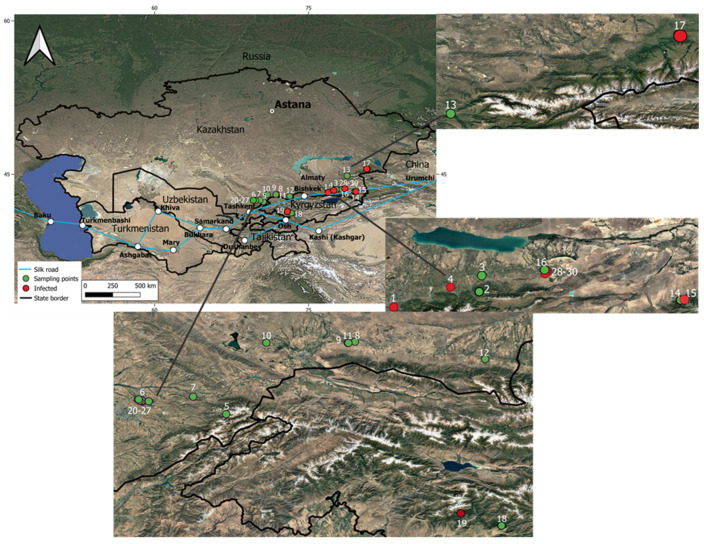
Geographic distribution of samples and infection foci of *Venturia inaequalis*. The main map shows sampling points (green) and locations where infection was detected (red). The historical route of the Silk Road is also indicated [23]. Insets provide detailed views of areas in Kazakhstan and Kyrgyzstan, with point numbering corresponding to sample identifiers. The map scale is indicated in kilometers.

Based on the survey results, a geospatial map was created displaying both the surveyed and infected apple populations (Figure 1).

**Figure 2:**
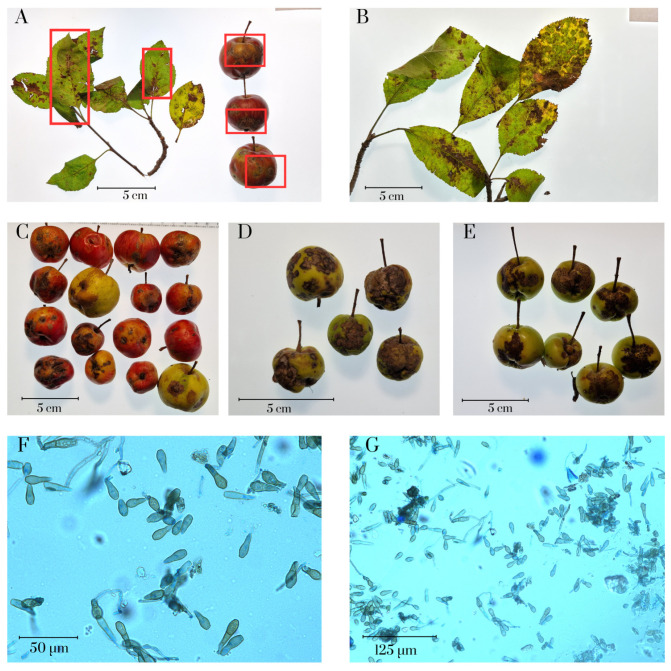
Images of plant samples infected with *Venturia inaequalis*. (**A**) the classic symptoms of apple scab are highlighted with red boxes. (**B**–**E**) images of plant samples; (**F**,**G**) microscopic images of *Venturia inaequalis*.e figure (**A**).

The symptoms observed on the surveyed plant samples matched previously described patterns [24,25]. On the fruits, multiple rounded or slightly oval, dark olive to brown, velvety spots of varying sizes, slightly sunken, were seen. The lesions ranged in size from small specks of 2–10 mm to complete fruit damage. As the infection progressed, the spots could merge, leading to extensive skin lesions. The affected areas exhibited a corky texture, resulting in noticeable structural changes in the fruit. Further disease development may cause cracking, creating conditions for secondary infections [2]. Overall, the fruit lesions we observed are consistent with previously reported observations [4,26]. As the infection advanced, the spots could merge, causing extensive damage to the fruit skin. On the leaves, brown to dark brown spots often developed, frequently with a thin dark margin along the edges. In severe cases, leaf blade deformation and drying of the tips and edges were noted. The observed pathological changes in the leaf lamina indicate a longstanding infection with mature sporulating structures and pronounced, severe sporulation [4]. Plant samples are shown in Figure 2A–E.

Conidia of *Venturia inaequalis* (Figure 2F,G) were oval or lemon-shaped. Their sizes ranged from 12–17 µm in length and 7–12 µm in width. Conidia were observed both with septa—mainly one, and rarely two—and without septa. The surface of the conidia was smooth, lacking distinctive reticulation or grooves. Previously published studies reported conidial sizes both consistent with our data [6,27] and larger [28]. The presence of septa is also a commonly observed feature [27]. These results confirm the characteristic morphological traits of *V. inaequalis* conidia.

However, while morphological analysis provides useful information in early diagnostic stages, it alone is insufficient for precise pathogen identification. Morphological features can vary based on environmental factors, colony age, and the fungus’s physiological state, making it difficult to differentiate closely related species accurately.

### 3.2. Molecular Identification of Venturia inaequalis and Phylogenetic Analysis

Based on PCR analysis, all examined samples were definitively identified as *Venturia inaequalis*. The amplification targeted the well-known marker gene *EF-1α*, commonly used for species-level diagnosis of phytopathogenic fungi. Each PCR reaction included a negative control. No amplification occurred in the negative controls, confirming the absence of contamination and the correctness of the PCR method. The Cq values showed moderate variability: mean = 27.96, median = 27.39, minimum = 20.82, maximum = 38.03. The standard deviation was 5.31, and the interquartile range was 10.09, reflecting the presence of both early and late amplifying samples. Several high Cq values (>36) probably indicated low template concentrations or partial DNA degradation. Amplification was absent in the negative control, further ensuring no contamination. To confirm the accuracy of identification, ITS region sequencing of ribosomal DNA was performed using the ONT platform. This approach allowed for unambiguous confirmation of *V. inaequalis* in the samples and excluded errors related to closely related species.

Sequencing results, applying a minimum quality threshold of Q ≥ 9, yielded 21.23 k reads that passed the quality filter and were used for downstream analysis. These reads served as the basis for constructing consensus sequences (File S1). ITS rRNA consensus sequences were generated by aligning reads against reference sequences and then analyzed with BLAST (https://blast.ncbi.nlm.nih.gov/Blast.cgi (accessed on 27 November 2025)) to determine phylogenetic relationships. Due to uneven coverage and alignment of the amplicon, ITS sequences were trimmed to 482 bp.

**Figure 3:**
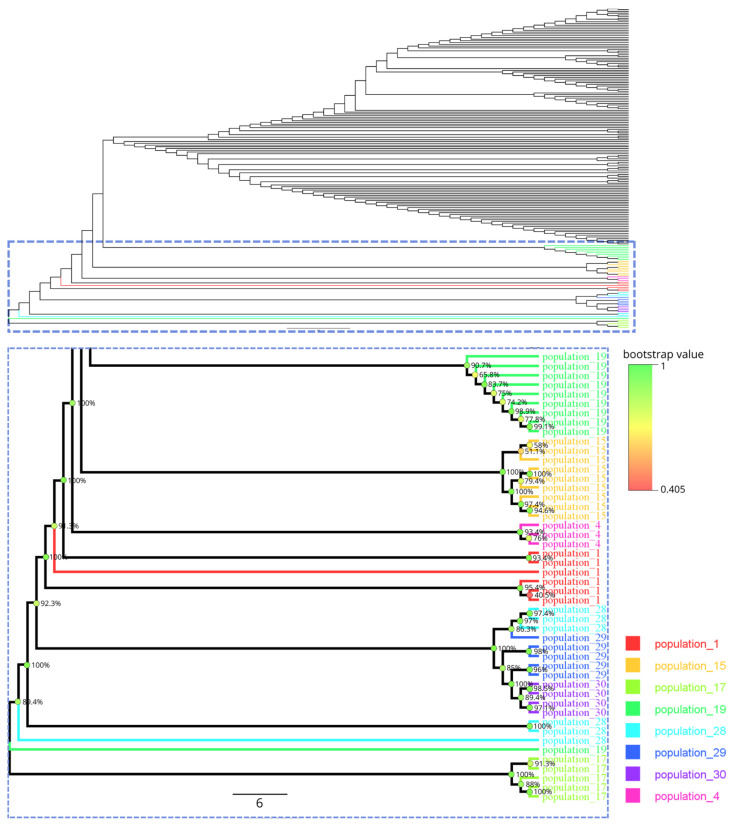
Phylogenetic tree of the studied isolates. Color coding corresponds to different populations. Branch lengths represent the genetic distance between samples. The enlarged fragment shows the details of population clustering, including the bootstrap values.

Phylogenetic analysis, based on 185 *Venturia inaequalis* sequences retrieved from the publicly available NCBI database, revealed high genetic diversity within the species (Figure 3). The Kyrgyz isolates formed a distinct cluster. In the phylogenetic tree, it was positioned next to a clade comprising isolates from international studies, indicating their phylogenetic relatedness. In contrast, isolates from Kazakhstan showed more pronounced genetic differentiation.

Kyrgyz samples (population 19, wild apple) formed a well-supported and clearly defined clade. On either side of this clade were isolates from India—Vi24 and Vi25—as well as samples from population 15 (wild apple from the Almaty region). A separate clade included samples from the Zhetysu region (population 17). Samples from the same population generally clustered closely and showed short intracluster branches, indicating low intrapopulation variability.

Overall, there is a clear trend toward the formation of geographically structured clusters, as well as clusters related to the degree of domestication of populations. The geographic association of such clusters has also been observed in other studies based on microsatellite analysis [8]. The phylogenetic tree reveals a sequential pattern: Zhetysu region—Almaty region—Kyrgyzstan—international populations. Isolates from Kazakhstan and Kyrgyzstan form separate clades, reflecting the influence of local environmental conditions, the genetic traits of the host apple, and possibly historical migration pathways of the pathogen. The proximity of the Kyrgyz isolates to international clusters suggests a potential influence of introduced cultivars or ancient connections between regions of Central and South Asia. This aligns with other studies showing the spread of *V. inaequalis* in apple domestication regions and subsequent migration alongside cultivated varieties [10]. The relative distance from the Kazakh isolates may be explained by natural barriers, such as the Tien Shan Mountains, as well as differences in agricultural history and the use of wild versus cultivated apples. This supports the hypothesis that pathogen populations from the native range of *M. sieversii* retain unique genetic lineages [8].

These findings confirm the presence of pronounced population structure in *V. inaequalis* and suggest the existence of geographic or ecological segregation among the studied lineages. However, a comprehensive understanding of evolutionary relationships requires analyses based on broader genomic coverage, including whole-genome sequencing data or multilocus genomic markers.

The data indicate that *Venturia inaequalis* remains a widely distributed phytopathogen in both cultivated and wild apple populations. The observed infection rate (16%) aligns with reports from regions with a moderately continental climate, where spring–summer humidity and temperature conditions favor scab development [27]. Notably, the detection of infection in wild *Malus sieversii* populations is significant, as these represent a valuable genetic resource and the primary center of origin of cultivated apple. The presence of the pathogen in these populations calls for close monitoring, as these foci may serve as reservoirs of infection and contribute to the emergence of new virulent strains. To reduce the impact of apple scab in practice, integrated management measures are applied, including the removal of fallen leaves to decrease sources of primary infection, the use of resistant apple cultivars, and timely fungicide applications during the peak release of ascospores. Obtaining accurate information on the presence of *V. inaequalis* in different types of orchards using molecular identification methods, such as sequencing and real-time PCR, enables more timely predictions of disease epidemics and optimization of plant protection measures. The results of our study provide a foundation for implementing targeted pathogen monitoring and developing locally adapted scab management strategies, contributing to reduced economic losses and improved fruit quality.

Ultimately, this study confirms that *V. inaequalis* populations in Central Asia are characterized by complex structure and heterogeneity, requiring integrated approaches for monitoring and predicting epidemiological dynamics. Increasing the sample size, including whole-genome sequencing data, and investigating environmental factors will enable a deeper understanding of pathogen adaptation and dispersal mechanisms, ultimately aiding in the development of more effective strategies to conserve the genetic diversity of wild apple populations and prevent scab outbreaks in regional orchards.

## 4. Conclusions

The study demonstrated that *Venturia inaequalis* is consistently present in both natural and cultivated apple populations in Kazakhstan and Kyrgyzstan, with an infection rate of 16%. Morphological analysis confirmed the characteristic scab symptoms on leaves and fruits; however, accurate identification required molecular methods, which were verified by *EF-1α* marker PCR and ITS region sequencing. Phylogenetic analysis revealed a clear geographic structure of populations, with distinct clades of Kazakh and Kyrgyz isolates, indicating local genetic differentiation. These findings highlight the importance of an integrated approach for monitoring and assessing the epiphytotic situation.

## Figures and Tables

**Table 1:** Infection severity scale values.

Value	Infection Severity
2	Less than 1% of leaves and fruits affected; symptoms are noticeable only upon close inspection
3	Up to 5% of affected organs, with no noticeable impact on the tree
4	Intermediate
5	Affected leaves and fruits are widely distributed, involving a significant portion of the tree, with approximately 25% infection severity
6	Intermediate
7	Severe infection: approximately 50% of organs heavily affected

**Table 2:** Primers used in the study.

Primer Design	Name	5′-3′ Sequence	Source
Real-time PCR analysis	F1	F: CACTTCCCCGCTATTCACGT	[17]
	R11	R: GCAATCGTTAGCATCGTCATAGTG	
	Ven1	[FAM]CTCAAGGCAGCCCAACTTTCTCCGGT[BHQ1]	
ONT sequencing	ITS4	F: TCCTCCGCTTATTGATATGC	[18]
	ITS5	R: GGAAGTAAAAGTCGTAACAAGG	

**Table 3:** Surveyed Apple Populations.

Garden Definition	Region	No. on Map	Sampling Location	Coordinates
Cultivated apple orchards	Almaty Region	1	Institute of Plant Biology and Biotechnology	43.2268, 76.91625
		2	v. Shelek	N/A
		3	v. Malovodnoye	43.51056, 77.70278
		4	v. Bolek	43.40639, 77.42306
	Turkestan Region	5	Bekzhan Farm	42.31667, 70.61667
		6	Shymkent, “Dala Fruit”	42.44023, 69.7849
		7	Tyulkubas District, “Almaly Sai”	42.49, 70.29
	Zhambyl Region	8	Taraz, “Ecofruit”	43.03519, 71.88858
		9	Taraz, “Alina Apple”	43.02272, 71.81756
		10	Taraz, “Auli Ata”	43.02607, 71.0131
		11	Taraz, “Grand apple”	43.02285, 71.82382
		12	v. Merke	42.86333, 73.17417
	Zhetysu Region	13	Tekeli	44.86306, 78.76417
Wild apple tree populations	Almaty Region	14	Sumbé	43.29306, 79.48417
		15	Ketpentau	43.30528, 79.75139
		16	Ile-Alatau State National Natural Park	43.36424, 77.68041
	Zhetysu Region	17	Zhongar-Alatau State National Natural Park	45.51746, 80.72224
	Kyrgyz Republic	18	v. Kara-Alma	41.210000953, 73.336669017
		19	Arslan Bob	41.333334292, 72.933335690
Abandoned cultivated apple orchards	Turkestan Region	20	Garden 1	42.44863821, 69.78857
		21	Garden 2	42.44779721, 69.79035526
		22	Garden 3	42.44968945, 69.78657079
		23	Garden 4	42.44874333, 69.76197175
		24	Garden 5	42.46041211, 69.74557239
		25	Garden 6	42.46230434, 69.75187984
		26	Garden 7	42.44522167, 69.84775302
		27	Garden 8	42.44401275, 69.85153749
	Almaty Region	28	Garden 11	43.55170546, 78.28706375
		29	Garden 12	43.55202083, 78.28900855
		30	Garden 13	43.54077255, 78.28506639

## Data Availability

The original contributions presented in this study are included in the article/Supplementary Material. Further inquiries can be directed to the corresponding author.

## Supplementary Materials

The following supporting information can be downloaded at: https://www.mdpi.com/article/10.3390/cimb47121011/s1.
