# Supplementary material for: Morphological and Molecular Characterization of Apple Scab (Venturia inaequalis) in Kazakhstan and Kyrgyzstan
Source: Curr Issues Mol Biol. 2025 Dec 2;47(12):1011. doi: 10.3390/cimb47121011 (PMC12731915; doi:10.3390/cimb47121011)
Supplement: Supplementary file 1 [file cimb-47-01011-s001.zip › cimb-4021410-supplementary.pdf]

Supplementary Materials: File S1. Sequences of ITS loci from *Venturia inaequalis* isolates obtained by target sequencing.

>p15_1
GTTAGCCTTCG-GGCGCAT-CCC-CACCCCCTG-TAACC--GCGGCCCGGATTCGGCG-CCTGGC-
GGGACCACCCCCCG
TTTGCGAGGGGCCCCGCCTGCCGGAATTAGCCAACCCTGCCTGGAAAATTGAAGTTTGAGA
ACAGCATCATTAGAACATA
AGTTTCAACAACGGATCTCTTGGCTCTCGCATCGATGAAGAACGCAGCGAAATGCGATAAG
TAATGTGAATTGCAGAATT
CAGTGAATCATCGAATCTTTGAACGCACCTTGCGCTCCTTGGTATTCCTTGGAGCATGCCTG
TTTGAGCGCCATTTCTAC
CCTGGAGCCCTGCTCT-
GTGATGGGCCCCGTCCTCGCGGACGGGCCCGAAACCCGTGGGCGCCGTCGTCCGGCCCTGA
GC
GTAGCAAGAGAAATCCCTCGCTCGGAGTGTCTGTCGGCCGGC-
CGCCCCGAAACCCATATTTA-CAAGGTTGGCCTCGGA
TC
>p15_2
GTTAGCCTTCG-GGCGCAT-CCC-CACCCCCTG-TAACC--GCGGCCCGGATTCGGCG-CCTGGC-
GGGACCACCCCCCG
TTTGCGAGGGGCCCCGCCTGCCGGAATTAGCCAACCCTGCCTGGAAAATTGAAGTTTGAGA
ACAGCATCATTAGAACATA
AGTTTCAACAACGGATCTCTTGGCTCTCGCATCGATGAAGAACGCAGCGAAATGCGATAAG
TAATGTGAATTGCAGAATT
CAGTGAATCATCGAATCTTTGAACGCACCTTGCGCTCCTTGGTATTCCTTGGAGCATGCCTG
TTTGAGCGCCATTTCTAC
CCTGGAGCCCTGCTCT-
GTGATGGGCCCCGTCCTCGCGGACGGGCCCGAAACCCGTGGGCGCCGTCGTCCGGCCCTGA
GC
GTAGCAAGAGAAATCCCTCGCTCGGAGTGTCTGTCGGCCGGC-
CGCCCCGAAACCCATATTTA-CAAGGTTGGCCTCGGA
TC
>p15_3
GTTAGCCTTCG-GGCGCAT-CCC-CACCCCCTG-TAACC--GCGGCCCGGATTCGGCG-CCTGGC-
GGGACCACCCCCCG
TTTGCGAGGGGCCCCGCCTGCCGGAATTAGCCAACCCTGCCTGGAAAATTGAAGTTTGAGA
ACAGCATCATTAGAACATA
AGTTTCAACAACGGATCTCTTGGCTCTCGCATCGATGAAGAACGCAGCGAAATGCGATAAG
TAATGTGAATTGCAGAATT
CAGTGAATCATCGAATCTTTGAACGCACCTTGCGCTCCTTGGTATTCCTTGGAGCATGCCTG
TTTGAGCGCCATTTCTAC
CCTGGAGCCCTGCTCT-
GTGATGGGCCCCGTCCTCGCGGACGGGCCCGAAACCCGTGGGCGCCGTCGTCCGGCCCTGA
GC
GTAGCAAGAGAAATCCCTCGCTCGGAGTGTCTGTCGGCCGGC-
CGCCCCGAAACCCATATTTA-CAAGGTTGGCCTCGGA
TC
>p15_4

GTTAGCCTTCG-GGCGCAT-CCC-CACCCCCTG-TAACC--GCGGCCCGGTTTCGGCG-CCTGGC-GGCACCACCCCCCG
TTTGCGAGGGGCCCCGCCTGCCGGAATTAGCCAACCCTGCCTGGAAAATTGAAGTTTGAGAACAGCATCATTAGAACATA
AGTTTCAACAACGGATCTCTTGGCTCTCGCATCGATGAAGAACGCAGCGAAATGCGATAAGTAATGTGAATTGCAGAATT
CAGTGAATCATCGAATCTTTGAACGCACCTTGCGCTCCTTGGTATTCCTTGGAGCATGCCTGTTTGAGCGCCATTTCTAC
CCTGGAGCCCTGCTCT-GTGATGGGCCCCGTCCTCGCGGACGGGCCCGAAACCCGTGGGCGCCGTCGTCCGGCCCTGAGC
GTAGCAAGAGAAATCCCTCGCTCGGAGTGTCTGTCGGCCGGC-CGCCCCGAAACCCATATTTA-CAAGGTTGGCCTCGGA
TC
>p15_5
GTTAGCCTTCG-GGCGCAT-CCC-CACCCCCTG-TAACC--GCGGCCCGGTTTCGGCG-CCTGGC-GGCACCACCCCCCG
TTTGCGAGGGGCCCCGCCTGCCGGAATTAGCCAACCCTGCCTGGAAAATTGAAGTTTGAGAACAGCATCATTAGAACATA
AGTTTCAACAACGGATCTCTTGGCTCTCGCATCGATGAAGAACGCAGCGAAATGCGATAAGTAATGTGAATTGCAGAATT
CAGTGAATCATCGAATCTTTGAACGCACCTTGCGCTCCTTGGTATTCCTTGGAGCATGCCTGTTTGAGCGCCATTTCTAC
CCTGGAGCCCTGCTCT-GTGATGGGCCCCGTCCTCGCGGACGGGCCCGAAACCCGTGGGCGCCGTCGTCCGGCCCTGAGC
GTAGCAAGAGAAATCCCTCGCTCGGAGTGTCTGTCGGCCGGC-CGCCCCGAAACCCATATTTA-CAAGGTTGGCCTCGGA
TC
>p15_6
GTTAGCCTTCG-GGCGCAT-CCC-CACCCCCTG-TAACC--GCGGCCCGGTTTCGGCG-CCTGGC-GGCACCACCCCCCG
TTTGCGAGGGGCCCCGCCTGCCGGAATTAGCCAACCCTGCCTGGAAAATTGAAGTTTGAGAACAGCATCATTAGAACATA
AGTTTCAACAACGGATCTCTTGGCTCTCGCATCGATGAAGAACGCAGCGAAATGCGATAAGTAATGTGAATTGCAGAATT
CAGTGAATCATCGAATCTTTGAACGCACCTTGCGCTCCTTGGTATTCCTTGGAGCATGCCTGTTTGAGCGCCATTTCTAC
CCTGGAGCCCTGCTCT-GTGATGGGCCCCGTCCTCGCGGACGGGCCCGAAACCCGTGGGCGCCGTCGTCCGGCCCTGAGC
GTAGCAAGAGAAATCCCTCGCTCGGAGTGTCTGTCGGCCGGC-CGCCCCGAAACCCATATTTA-CAAGGTTGGCCTCGGA
TC
>p15_7
ATTAGCCTTCG-GGCGCAT-CCC-CACCCCCTG-TAACC--GCGGCCCGGTTTCGGCG-CCTGGC-GGGACCACCCCCCG
TTTGCGAGGGGCCCCGCCTGCCGGAATTAGCCAACCCTGCCTGGAAAATTGAAGTTTGAGAACAGCATCATTAGAACATA
AGTTTCAACAACGGATCTCTTGGCTCTCGCATCGATGAAGAACGCAGCGAAATGCGATAAGTAATGTGAATTGCAGAATT

CAGTGAATCATCGAATCTTTGAACGCACCTTGCGCTCCTTGGTATTCCTTGGAGCATGCCTG
TTTGAGCGCCATTTCTAC
CCTGGAGCCCTGCTCT-
GTGATGGGCCCCGTCCTCGCGGACGGGCCCGAAACCCGTGGGCGCCGTCGTCCGGCCCTGA
GC
GTAGCAAGAGAAATCCCTCGCTCGGAGTGTCTGTCGGCCGGC-
CGCCCCGAAACCCATATTTA-CAAGGTTGGCCTCGGA
TC
>p15_8
ATTAGCCTTCG-GGCGCAT-CCC-CACCCCCTG-TAACC--GCGGCCCGGTTTCGGCG-CCTGGC-
GGGACCACCCCCCG
TTTGCGAGGGGCCCCGCCTGCCGGAATTAGCCAACCCTGCCTGGAAAATTGAAGTTTGAGA
ACAGCATCATTAGAACATA
AGTTTCAACAACGGATCTCTTGGCTCTCGCATCGATGAAGAACGCAGCGAAATGCGATAAG
TAATGTGAATTGCAGAATT
CAGTGAATCATCGAATCTTTGAACGCACCTTGCGCTCCTTGGTATTCCTTGGAGCATGCCTG
TTTGAGCGCCATTTCTAC
CCTGGAGCCCTGCTCT-
GTGATGGGCCCCGTCCTCGCGGACGGGCCCGAAACCCGTGGGCGCCGTCGTCCGGCCCTGA
GC
GTAGCAAGAGAAATCCCTCGCTCGGAGTGTCTGTCGGCCGGC-
CGCCCCGAAACCCATATTTA-CAAGGTTGGCCTCGGA
TC
>p15_9
ATTAGCCTTCG-GGCGCAT-CCC-CACCCCCTG-TAACC--GCGGCCCGGTTTCGGCG-CCTGGC-
GGGACCACCCCCCG
TTTGCGAGGGGCCCCGCCTGCCGGAATTAGCCAACCCTGCCTGGAAAATTGAAGTTTGAGA
ACAGCATCATTAGAACATA
AGTTTCAACAACGGATCTCTTGGCTCTCGCATCGATGAAGAACGCAGCGAAATGCGATAAG
TAATGTGAATTGCAGAATT
CAGTGAATCATCGAATCTTTGAACGCACCTTGCGCTCCTTGGTATTCCTTGGAGCATGCCTG
TTTGAGCGCCATTTCTAC
CCTGGAGCCCTGCTCT-
GTGATGGGCCCCGTCCTCGCGGACGGGCCCGAAACCCGTGGGCGCCGTCGTCCGGCCCTGA
GC
GTAGCAAGAGAAATCCCTCGCTCGGAGTGTCTGTCGGCCGGC-
CGCCCCGAAACCCATATTTA-CAAGGTTGGCCTCGGA
TC
>p1_1
GTTAGCCTTCG-GGCGCAT-CCC-CACCCTCTG-TAACC--GCGGCCCGGATTCGGCG-CCTGGC-
GGGACCACCCCCCG
TTTGCGAGGGGGCCCGCCTGCCGGAATTAGCCAACCCTGCCTGGAAAATTGAAGTCTGAGA
ACAAGTTAAATAAAACAAA
ACTTTCAACAACGGATCTCTTGGTTCTGGCATCGATGAAGAACGCAGCGAAATGCGATAAG
TAATGTGAATTGCAGAATT
CAGTGAATCATCGAATCTTTGAACGCACATTGCGCCCCCTGGTATTCCGGGGGGCACGCCT
GTTCGAGCGTCATTTCTAC
CCTCAAGCCCTGCTCT-
GTGATGGGCCCCGTCCTCGCGGACGGGCCCGAAACCCGTGGGCGCCGTCGTCCGGCCCTGA
GC

GTAGCAAGAGAAATCCCTCGCTCGGAGTGTCTGTCGGCAGGC-
CGCCCCGAAACCCATATTTA-CAAGGTTGACCTCGGA
TC
>p1_2
GTTAGCCTTCG-GGCGCAT-CCC-CACCCTCTG-TAACC--GCGGCCCGGATTCGGCG-CCTGGC-
GGGACCACCCCCCG
TTTGCGAGGGGGCCCGCCTGCCGGAATTAGCCAACCCTGCCTGGAAAATTGAAGTCTGAGA
ACAAGTTAAATAAAACAAA
ACTTTCAACAACGGATCTCTTGGTTCTGGCATCGATGAAGAACGCAGCGAAATGCGATAAG
TAATGTGAATTGCAGAATT
CAGTGAATCATCGAATCTTTGAACGCACATTGCGCCCCCTGGTATTCCGGGGGGCACGCCT
GTTCGAGCGTCATTTCTAC
CCTCAAGCCCTGCTCT-
GTGATGGGCCCCGTCCTCGCGGACGGGCCCGAAACCCGTGGGCGCCGTCGTCCGGCCCTGA
GC
GTAGCAAGAGAAATCCCTCGCTCGGAGTGTCTGTCGGCAGGC-
CGCCCCGAAACCCATATTTA-CAAGGTTGACCTCGGA
TC
>p1_3
GTTAGCCTTCG-GGCGCAT-CCC-CACCCTCTG-TAACC--GCGGCCCGGATTCGGCG-CCTGGC-
GGGACCACCCCCCG
TTTGCGAGGGGGCCCGCCTGCCGGAATTAGCCAACCCTGCCTGGAAAATTGAAGTCTGAGA
ACAAGTTAAATAAAACAAA
ACTTTCAACAACGGATCTCTTGGTTCTGGCATCGATGAAGAACGCAGCGAAATGCGATAAG
TAATGTGAATTGCAGAATT
CAGTGAATCATCGAATCTTTGAACGCACATTGCGCCCCCTGGTATTCCGGGGGGCACGCCT
GTTCGAGCGTCATTTCTAC
CCTCAAGCCCTGCTCT-
GTGATGGGCCCCGTCCTCGCGGACGGGCCCGAAACCCGTGGGCGCCGTCGTCCGGCCCTGA
GC
GTAGCAAGAGAAATCCCTCGCTCGGAGTGTCTGTCGGCAGGC-
CGCCCCGAAACCCATATTTA-CAAGGTTGACCTCGGA
TC
>p29_1
GTTAGCCTTCG-GGCGCAT-CCC-CACCCCCTG-TAACC--GTTGCCCGGATTCGGCG-CCTGGC-
GGGACCACCCTCCG
TTTGCGAGGGGCCCCGCCTGCCGGAATTAGCCAACCCTGCCTGGAAAATTGCAGTCTGAGA
ACAACTTAAATAAATTAAA
ACTTTCAACAACGGATCTCTTGGTTCTGGCATCGATGAAGAACGCAGCGAAATGCGATAAG
TAATGTGAATTGCAGAATT
CAGTGAATCATCGAATCTTTGAACGCACATTGCGCCCCTTGGTATTCCGGGGGGCATGCCTG
TTCGAGCGTCATTTCTAC
CCTCAAGCCTTGCTTG-
GTGTTGGGCCGCGTCCTCGCGGACGTGCCTTAAACCCGTGGGCGCCGTCGTCTGGCCCTAA
GC
GTAGCAAGAGAAATCACTCGCTCGGAGTGTCTGTCGGCAGGC-
CGCCCTAAAACCCATATTTA-CAAGGTTGACCTCGGA
TC
>p28_1
GTTAGCCTTCG-GGCGCAT-CCC-CAACCCGTG-TAACC--GCGGCCCGGATTCGGCG-
CCTGGC-GGGGCCACCCCCCG

TTTGCGCGGGGCCCCGCCTGCCGGAATTAGCCAACCCTGCCTGGAAAATTGCAGTCTGAGA
ACAACTTAAATAAATCAAA
ACTTTCAACAACGGATCTCTTGGTTCTGGCATCGATGAAGAACGCAGCGAAATGCGATAAG
TAATGTGAATTGCAGAATT
CAGTGAATCATCGAATCTTTGAACGCACATTGCGCCCCCTGGTATTCCGGGGGGCATGCCT
GTTCGAGCGTCATTTCTAC
CCTCAAGCCTTGCTTG-
GTGTTGGGCCGCGTCCTCGCGGACGTGCCCTAAACCCGTGGGCGCCGTCGTCCGGCCCTGA
GC
GTAGCAAGAGAAATCACTCGCTCGGAGTGTCTGTCGGCAGGC-
CGCCCCGAAACCCATATTTA-CAAGGTTGACCTCGGA
TC
>p28_2
GTTAGCCTTCG-GGCGCAT-CCC-CAACCCGTG-TAACC--GCGGCCCGGATTCGGCG-
CCTGGC-GGGGCCACCCCCCG
TTTGCGCGGGGCCCCGCCTGCCGGAATTAGCCAACCCTGCCTGGAAAATTGCAGTCTGAGA
ACAACTTAAATAAATCAAA
ACTTTCAACAACGGATCTCTTGGTTCTGGCATCGATGAAGAACGCAGCGAAATGCGATAAG
TAATGTGAATTGCAGAATT
CAGTGAATCATCGAATCTTTGAACGCACATTGCGCCCCCTGGTATTCCGGGGGGCATGCCT
GTTCGAGCGTCATTTCTAC
CCTCAAGCCTTGCTTG-
GTGTTGGGCCGCGTCCTCGCGGACGTGCCCTAAACCCGTGGGCGCCGTCGTCCGGCCCTGA
GC
GTAGCAAGAGAAATCACTCGCTCGGAGTGTCTGTCGGCAGGC-
CGCCCCGAAACCCATATTTA-CAAGGTTGACCTCGGA
TC
>p28_3
GTTAGCCTTCG-GGCGCAT-CCC-CAACCCGTG-TAACC--GCGGCCCGGATTCGGCG-
CCTGGC-GGGGCCACCCCCCG
TTTGCGCGGGGCCCCGCCTGCCGGAATTAGCCAACCCTGCCTGGAAAATTGCAGTCTGAGA
ACAACTTAAATAAATCAAA
ACTTTCAACAACGGATCTCTTGGTTCTGGCATCGATGAAGAACGCAGCGAAATGCGATAAG
TAATGTGAATTGCAGAATT
CAGTGAATCATCGAATCTTTGAACGCACATTGCGCCCCCTGGTATTCCGGGGGGCATGCCT
GTTCGAGCGTCATTTCTAC
CCTCAAGCCTTGCTTG-
GTGTTGGGCCGCGTCCTCGCGGACGTGCCCTAAACCCGTGGGCGCCGTCGTCCGGCCCTGA
GC
GTAGCAAGAGAAATCACTCGCTCGGAGTGTCTGTCGGCAGGC-
CGCCCCGAAACCCATATTTA-CAAGGTTGACCTCGGA
TC
>p29_2
GTTAGCCTTCG-GGCGCAT-CCC-CACCCCGTG-TAACT--GCGTCCCGGATTCGGTG-CCTGGC-
GGGCCCACCCCCCG
TTTGCGAGGGGCTCCGCCTGCCGGAATTATCCAACCCTGCCTTGAAAATTGAAGTCTGAGA
AAAACTTAAATAAATTAAA
ACTTTCAACAACGGATCTCTTGGTTCTGGCATCGATGAAGAACGCAGCGAAATGCGATAAG
TAATGTGAATTGCAGAATT
CAGTGAATCATCGAATCTTTGAACGCACATTGCGCCCCTTGGTATTCCGAGGGGCATGCCTG
TTCGAGCGTCATTTGTAC

CCTCAAGCCCTGCTTG-
GTGTTGGGCCTCGTCCTCGCGGACGCGCCTGAAACCAATGGGCGCCGTCGTCTGGCCCTGA
GC
GTAGCAAGAGAAATCCCTCGCTCGAAGTGTCTATCGGCAGGC-
CGCCCAAAAACCCATATTTA-CAAGGTTGACCTCGGA
TC
>p29_3
GTTAGCCTTCG-GGCGCAT-CCC-CACCCCGTG-TAACT--GCGTCCCGGATTCGGTG-CCTGGC-
GGGCCCACCCCCCG
TTTGCGAGGGGCTCCGCCTGCCGGAATTATCCAACCCTGCCTTGAAAATTGAAGTCTGAGA
AAAACTTAAATAAATTAAA
ACTTTCAACAACGGATCTCTTGGTTCTGGCATCGATGAAGAACGCAGCGAAATGCGATAAG
TAATGTGAATTGCAGAATT
CAGTGAATCATCGAATCTTTGAACGCACATTGCGCCCCTTGGTATTCCGAGGGGCATGCCTG
TTCGAGCGTCATTTGTAC
CCTCAAGCCCTGCTTG-
GTGTTGGGCCTCGTCCTCGCGGACGCGCCTGAAACCAATGGGCGCCGTCGTCTGGCCCTGA
GC
GTAGCAAGAGAAATCCCTCGCTCGAAGTGTCTATCGGCAGGC-
CGCCCAAAAACCCATATTTA-CAAGGTTGACCTCGGA
TC
>p29_4
GTTAGCCTTCG-GGCGCAT-CCC-CACCCCGTG-TAACT--GCGTCCCGGATTCGGTG-CCTGGC-
GGGCCCACCCCCCG
TTTGCGAGGGGCTCCGCCTGCCGGAATTATCCAACCCTGCCTTGAAAATTGAAGTCTGAGA
AAAACTTAAATAAAATACA
ACTTTCAACAACGGATCTCTTGGTTCTGGCATCGATGAAGAACGCAGCGAAATGCGATAAG
TAATGTGAATTGCAGAATT
CAGTGAATCATCGAATCTTTGAACGCACATTGCGCCCCTTGGTATTCCGAGGGGCATGCCTG
TTCGAGCGTCATTTGTAC
CCTCAAGCTCTGCTTG-
GTGTTGGGCCTCGTCCTCGCGGACGCGCCCGAAACCAATGGGCGCCGTCGTCTGGCCCTGA
GC
GTAGCAAGAGAAATCCCTCGCTCGAAGTGTCTATCGGCAGGC-
CGCCCAAAAACCCATATTTA-CAAGTTTGACCTCGGA
TC
>p29_5
GTTAGCCTTCG-GGCGCAT-CCC-CACCCCGTG-TAACT--GCGTCCCGGATTCGGTG-CCTGGC-
GGGCCCACCCCCCG
TTTGCGAGGGGCTCCGCCTGCCGGAATTATCCAACCCTGCCTTGAAAATTGAAGTCTGAGA
AAAACTTAAATAAAATACA
ACTTTCAACAACGGATCTCTTGGTTCTGGCATCGATGAAGAACGCAGCGAAATGCGATAAG
TAATGTGAATTGCAGAATT
CAGTGAATCATCGAATCTTTGAACGCACATTGCGCCCCTTGGTATTCCGAGGGGCATGCCTG
TTCGAGCGTCATTTGTAC
CCTCAAGCTCTGCTTG-
GTGTTGGGCCTCGTCCTCGCGGACGCGCCCGAAACCAATGGGCGCCGTCGTCTGGCCCTGA
GC
GTAGCAAGAGAAATCCCTCGCTCGAAGTGTCTATCGGCAGGC-
CGCCCAAAAACCCATATTTA-CAAGTTTGACCTCGGA
TC

>p30_2
GTTAGCCTTCG-GGTGCAT-CTC-CAACCCGTG-TAACT--GCGGCCCGTATTCGTTT-CCTGGC-GGGTCCACCCGCCG
TTTGCGAGGGGCTCCGCCTGCCGGAATTATCCAACCCTGCGTTGAAAATTGAAGTCTGAGAAAAACTTAAATAAAATACA
ACTTTCAACAACGGATCTCTTGGTTCTCGCATCGATGAAGAACGCAGCGAAATGCGATAAGTAATGTGAATTGCAGAATT
CAGTGAATCATCGAATCTTTGAACGCACCTTGCGCCCCTTGGTATTCCGAGGGGCATGCCTGTTTGAGCGTCATTTGTAC
CCTCAAGCTCTGCTTG-GTGTTGGGTCTCGTCCTCGCGGACTCGCCCAAAACAATTGGGCGCCGGCGTATGGCTCTGAGC
GTAGCCAGATAAATCTCTCGCTTGCAGTGTCTATCGGCAGGA-CGCCCAAAAACCCATATTTA-CAATCTTGACCTCGGA
TC
>p30_1
GTTAGCCTTCG-GGTGCAT-CTC-CAACCCGTG-TAACT--GCGGCCCGTATTCGTTT-CCTGGC-GGGTCCACCCGCCG
TTTGCGAGGGGCTCCGCCTGCCGGAATTATCCAACCCTGCGTTGAAAATTGAAGTCTGAGAAAAACTTAAATAAAATACA
ACTTTCAACAACGGATCTCTTGGTTCTCGCATCGATGAAGAACGCAGCGAAATGCGATAAGTAATGTGAATTGCAGAATT
CAGTGAATCATCGAATCTTTGAACGCACCTTGCGCCCCTTGGTATTCCGAGGGGCATGCCTGTTTGAGCGTCATTTGTAC
CCTCAAGCTCTGCTTG-GTGTTGGGTCTCGTCCTCGCGGACTCGCCCAAAACAATTGGGCGCCGGCGTATGGCTCTGAGC
GTAGCCAGATAAATCTCTCGCTTGCAGTGTCTATCGGCAGGA-CGCCCAAAAACCCATATTTA-CAATCTTGACCTCGGA
TC
>p30_3
GTTAGCCTTCG-GGCGCAT-CCC-CAACCCATG-TAACT--GCGGCCCGGATTCGTTG-CCTGGC-GGGTCCACCCGCCG
TTTGCGAGGGGCTCCGCCTGCCGGAATTATCCAACCCTGCGTTGAAAATTGCAGTCTGAGAACAACTTAAATAAAATACA
ACTTTCAACAACGGATCTCTTGGTTCTCGCATCGATGAAGAACGCAGCGAAATGCGATAAGTAATGTGAATTGCAGAATT
CAGTGAATCATCGAATCTTTGAACGCACCTTGCGCCCCTTGGTATTCCGAGGGGCATGCCTGTTTGAGCGTCATTTGTAC
CCTCAAGCTCTGCTTG-GTGTTGGGTCTCGGCCTCGCGGACTCGCCCAAAACAATTGGGCGCCGGCGTATGGCTCTAAGC
GTAGCCAGATAAATCTCTCGCTTGCAGTGTCTATCGGCAGGC-CGCCCAAAAACCCATATTTA-CAATTTTGACCTCGGA
TC
>p30_4
GTTAGCCTTCG-GGCGCAT-CCC-CAACCCATG-TAACT--GCGGCCCGGATTCGTTG-CCTGGC-GGGTCCACCCGCCG
TTTGCGAGGGGCTCCGCCTGCCGGAATTATCCAACCCTGCGTTGAAAATTGCAGTCTGAGAACAACTTAAATAAAATACA

ACTTTCAACAACGGATCTCTTGGTTCTCGCATCGATGAAGAACGCAGCGAAATGCGATAAG
TAATGTGAATTGCAGAATT
CAGTGAATCATCGAATCTTTGAACGCACCTTGCGCCCCTTGGTATTCCGAGGGGCATGCCTG
TTTGAGCGTCATTTGTAC
CCTCAAGCTCTGCTTG-
GTGTTGGGTCTCGGCCTCGCGGACTCGCCCAAAACAATTGGGCGCCGGCGTATGGCTCTAA
GC
GTAGCCAGATAAATCTCTCGCTTGCAGTGTCTATCGGCAGGC-
CGCCCAAAAACCCATATTTA-CAATTTTGACCTCGGA
TC
>p1_4
GTTAGCCTTCG-GGCGCAT-CCC-CACCCTCTG-TAACC--GCGGCCCGGATTCGGCG-CCTGGC-
GGGACCACCCCCCG
TTTGCGAGGGGCCCCGCCTGCCGGAATTAGCCAACCCTGCCTGGAAAATTGAAGTCTGAGA
ACAAGTTAAATAAAACAAA
ACTTTCAACAACGGATCTCTTGGTTCTGGCATCGATGAAGAACGCAGCGAAATGCGATAAG
TAATGTGAATTGCAGAATT
CAGTGAATCATCGAATCTTTGAACGCACATTGCGCCCCCTGGTATTCCGGGGGGCACGCCT
GTTCGAGCGTCATTTCTAC
CCTGAAGCCCTGCTCT-
GTGATGGGCCCCGTCCTCGCGGACGGGCCCGAAACCCGTGGGCGCCGTCGTCCGGCCCTGA
GC
GTAGCAAGAGAAATCCCTCGCTCGGAGTGTCTGTCGGCAGGC-
CGCCCCGAAACCCATATTTA-CAAGGTTGGCCTCGGA
TC
>p1_5
GTTAGCCTTCG-GGCGCAT-CCC-CACCCTCTG-TAACC--GCGGCCCGGATTCGGCG-CCTGGC-
GGGACCACCCCCCG
TTTGCGAGGGGCCCCGCCTGCCGGAATTAGCCAACCCTGCCTGGAAAATTGAAGTCTGAGA
ACAAGTTAAATAAAACAAA
ACTTTCAACAACGGATCTCTTGGTTCTGGCATCGATGAAGAACGCAGCGAAATGCGATAAG
TAATGTGAATTGCAGAATT
CAGTGAATCATCGAATCTTTGAACGCACATTGCGCCCCCTGGTATTCCGGGGGGCACGCCT
GTTCGAGCGTCATTTCTAC
CCTGAAGCCCTGCTCT-
GTGATGGGCCCCGTCCTCGCGGACGGGCCCGAAACCCGTGGGCGCCGTCGTCCGGCCCTGA
GC
GTAGCAAGAGAAATCCCTCGCTCGGAGTGTCTGTCGGCAGGC-
CGCCCCGAAACCCATATTTA-CAAGGTTGGCCTCGGA
TC
>p1_6
GTTAGCCTTCG-GGCGCAT-CCC-CACCCTCTG-TAACC--GCGGCCCGGATTCGGCG-CCTGGC-
GGGACCACCCCCCG
TTTGCGAGGGGCCCCGCCTGCCGGAATTAGCCAACCCTGCCTGGAAAATTGAAGTCTGAGA
ACAAGTTAAATAAAACAAA
ACTTTCAACAACGGATCTCTTGGTTCTGGCATCGATGAAGAACGCAGCGAAATGCGATAAG
TAATGTGAATTGCAGAATT
CAGTGAATCATCGAATCTTTGAACGCACATTGCGCCCCCTGGTATTCCGGGGGGCACGCCT
GTTCGAGCGTCATTTCTAC

CCTGAAGCCCTGCTCT-
GTGATGGGCCCCGTCCTCGCGGACGGGCCCGAAACCCGTGGGCGCCGTCGTCCGGCCCTGA
GC
GTAGCAAGAGAAATCCCTCGCTCGGAGTGTCTGTCGGCAGGC-
CGCCCCGAAACCCATATTTA-CAAGGTTGGCCTCGGA
TC
>p19_1
GTTAACCTGCC-GGCAGAA-CCC-CAACCCGTT-TAACC--GCGGGTCGGATTCGGCG-
CCTGGCCGGGCCT-CCCCCCG
TTCGCCCGGGAGCCCGCCTCCCGGAATTAGCCAACCCTGCCTGGAAAATTGAAGTCTGAGA
ACAAGTTAAATAAGTCAAA
ACTCTCGGCAACGGATATCTCGGCTCTCGCATCGATGAAGAACGTAGCGAAATGCGATACT
TGGTGTGAATTGCAGAATC
CCGTGAACCATCGAGTCTTTGAACGCAAGTTGCGCCCGAAGCCATTCAGGGGGGCACGCCT
GCCTGGGCGTCATTTCTAC
CCTGGAGCCCTGCTCT-
GTGATGGGCCCCGTCCTCGCGGACGGGCCCGAAACCCGTGGGCGCCGTCGTCCGGCCCTGA
GC
GTAGCAAGAGAAATCCCTCGCTCGGAGTGTCTGTCGGCAGGC-
CGCCCCGAAACCCATATTTA-CAAGGTTGGCCTCGGA
TC
>p19_1 1
GTTAGCCTTCG-GGCGCAT-CCC-CACCCTCTG-TAACC--GCGGCCCGGATTCGGCG-CCTGGC-
GGGACCACCCCCCG
TTTGCGAGGGGCCCCGCCTGCCGGAATTAGCCAACCCTGCCTGGAAAATTGAAGTCTGAGA
ACAAGTTAAATAAAACAAA
ACTTTCAACAACGGATCTCTTGGTTCTGGCATCGATGAAGAACGCAGCGAAATGCGATAAG
TAATGTGAATTGCAGAATT
CAGTGAATCATCGAATCTTTGAACGCACATTGCGCCCCCTGGTATTCCGGGGGGCACGCCT
GTTCGAGCGCCATTTCTAC
CCTGGAGCCCTGCTCT-
GTGATGGGCCCCGTCCTCGCGGACGGGCCCGAAACCCGTGGGCGCCGTCGTCCGGCCCTGA
GC
GTAGCAAGAGAAATCCCTCGCTCGGAGTGTCTGTCGGCAGGC-
CGCCCCGAAACCCATATTTA-CAAGGTTGGCCTCGGA
TC
>p19_2
GTTAGCCTTCG-GGCGCAT-CCC-CACCCTCTG-TAACC--GCGGCCCGGATTCGGCG-CCTGGC-
GGGACCACCCCCCG
TTTGCGAGGGGCCCCGCCTGCCGGAATTAGCCAACCCTGCCTGGAAAATTGAAGTCTGAGA
ACAAGTTAAATAAAACAAA
ACTTTCAACAACGGATCTCTTGGTTCTGGCATCGATGAAGAACGCAGCGAAATGCGATAAG
TAATGTGAATTGCAGAATT
CAGTGAATCATCGAATCTTTGAACGCACATTGCGCCCCCTGGTATTCCGGGGGGCACGCCT
GTTCGAGCGCCATTTCTAC
CCTGGAGCCCTGCTCT-
GTGATGGGCCCCGTCCTCGCGGACGGGCCCGAAACCCGTGGGCGCCGTCGTCCGGCCCTGA
GC
GTAGCAAGAGAAATCCCTCGCTCGGAGTGTCTGTCGGCAGGC-
CGCCCCGAAACCCATATTTA-CAAGGTTGGCCTCGGA
TC

>p19_3
GTTAGCCTTCG-GGCGCAT-CCC-CACCCTCTG-TAACC--GCGGCCCGGATTCGGCG-CCTGGC-
GGGACCACCCCCCG
TTTGCGAGGGGCCCCGCCTGCCGGAATTAGCCAACCCTGCCTGGAAAATTGAAGTCTGAGA
ACAAGTTAAATAAAACAAA
ACTTTCAACAACGGATCTCTTGGTTCTGGCATCGATGAAGAACGCAGCGAAATGCGATAAG
TAATGTGAATTGCAGAATT
CAGTGAATCATCGAATCTTTGAACGCACATTGCGCCCCCTGGTATTCCGGGGGGCACGCCT
GTTCGAGCGCCATTTCTAC
CCTGGAGCCCTGCTCT-
GTGATGGGCCCCGTCCTCGCGGACGGGCCCGAAACCCGTGGGCGCCGTCGTCCGGCCCTGA
GC
GTAGCAAGAGAAATCCCTCGCTCGGAGTGTCTGTCGGCAGGC-
CGCCCCGAAACCCATATTTA-CAAGGTTGGCCTCGGA
TC
>p19_4
GTTAGCCTTCG-GGCGCAT-CCC-CACCCTCTG-TAACC--GCGGCCCGGATTCGGCG-CCTGGC-
GGGACCACCCCCCG
TTTGCGAGGGGCCCCGCCTGCCGGAATTAGCCAACCCTGCCTGGAAAATTGAAGTCTGAGA
ACAAGTTAAATAAAACAAA
ACTTTCAACAACGGATCTCTTGGTTCTGGCATCGATGAAGAACGCAGCGAAATGCGATAAG
TAATGTGAATTGCAGAATT
CAGTGAATCATCGAATCTTTGAACGCACATTGCGCCCCCTGGTATTCCGGGGGGCACGCCT
GTTCGAGCGCCATTTCTAC
CCTGGAGCCCTGCTCT-
GTGATGGGCCCCGTCCTCGCGGACGGGCCCGAAACCCGTGGGCGCCGTCGTCCGGCCCTGA
GC
GTAGCAAGAGAAATCCCTCGCTCGGAGTGTCTGTCGGCAGGC-
CGCCCCGAAACCCATATTTA-CAAGGTTGGCCTCGGA
TC
>p19_5
GTTAGCCTTCG-GGCGCAT-CCC-CACCCTCTG-TAACC--GCGGCCCGGATTCGGCG-CCTGGC-
GGGACCACCCCCCG
TTTGCGAGGGGCCCCGCCTGCCGGAATTAGCCAACCCTGCCTGGAAAATTGAAGTCTGAGA
ACAAGTTAAATAAAACAAA
ACTTTCAACAACGGATCTCTTGGTTCTGGCATCGATGAAGAACGCAGCGAAATGCGATAAG
TAATGTGAATTGCAGAATT
CAGTGAATCATCGAATCTTTGAACGCACATTGCGCCCCCTGGTATTCCGGGGGGCACGCCT
GTTCGAGCGCCATTTCTAC
CCTGGAGCCCTGCTCT-
GTGATGGGCCCCGTCCTCGCGGACGGGCCCGAAACCCGTGGGCGCCGTCGTCCGGCCCTGA
GC
GTAGCAAGAGAAATCCCTCGCTCGGAGTGTCTGTCGGCAGGC-
CGCCCCGAAACCCATATTTA-CAAGGTTGGCCTCGGA
TC
>p19_6
GTTAGCCTTCG-GGCGCAT-CCC-CACCCTCTG-TAACC--GCGGCCCGGATTCGGCG-CCTGGC-
GGGACCACCCCCCG
TTTGCGAGGGGCCCCGCCTGCCGGAATTAGCCAACCCTGCCTGGAAAATTGAAGTCTGAGA
ACAAGTTAAATAAAACAAA

ACTTTCAACAACGGATCTCTTGGTTCTGGCATCGATGAAGAACGCAGCGAAATGCGATAAG
TAATGTGAATTGCAGAATT
CAGTGAATCATCGAATCTTTGAACGCACATTGCGCCCCCTGGTATTCCGGGGGGCACGCCT
GTTCGAGCGCCATTTCTAC
CCTGGAGCCCTGCTCT-
GTGATGGGCCCCGTCCTCGCGGACGGGCCCGAAACCCGTGGGCGCCGTCGTCCGGCCCTGA
GC
GTAGCAAGAGAAATCCCTCGCTCGGAGTGTCTGTCGGCAGGC-
CGCCCCGAAACCCATATTTA-CAAGGTTGGCCTCGGA
TC
>p19_7
GTTAGCCTTCG-GGCGCAT-CCC-CACCCTCTG-TAACC--GCGGCCCGGATTCGGCG-CCTGGC-
GGGACCACCCCCCG
TTTGCGAGGGGCCCCGCCTGCCGGAATTAGCCAACCCTGCCTGGAAAATTGAAGTCTGAGA
ACAAGTTAAATAAAACAAA
ACTTTCAACAACGGATCTCTTGGTTCTGGCATCGATGAAGAACGCAGCGAAATGCGATAAG
TAATGTGAATTGCAGAATT
CAGTGAATCATCGAATCTTTGAACGCACATTGCGCCCCCTGGTATTCCGGGGGGCACGCCT
GTTCGAGCGCCATTTCTAC
CCTGGAGCCCTGCTCT-
GTGATGGGCCCCGTCCTCGCGGACGGGCCCGAAACCCGTGGGCGCCGTCGTCCGGCCCTGA
GC
GTAGCAAGAGAAATCCCTCGCTCGGAGTGTCTGTCGGCAGGC-
CGCCCCGAAACCCATATTTA-CAAGGTTGGCCTCGGA
TC
>p19_8
GTTAGCCTTCG-GGCGCAT-CCC-CACCCTCTG-TAACC--GCGGCCCGGATTCGGCG-CCTGGC-
GGGACCACCCCCCG
TTTGCGAGGGGCCCCGCCTGCCGGAATTAGCCAACCCTGCCTGGAAAATTGAAGTCTGAGA
ACAAGTTAAATAAAACAAA
ACTTTCAACAACGGATCTCTTGGTTCTGGCATCGATGAAGAACGCAGCGAAATGCGATAAG
TAATGTGAATTGCAGAATT
CAGTGAATCATCGAATCTTTGAACGCACATTGCGCCCCCTGGTATTCCGGGGGGCACGCCT
GTTCGAGCGCCATTTCTAC
CCTGGAGCCCTGCTCT-
GTGATGGGCCCCGTCCTCGCGGACGGGCCCGAAACCCGTGGGCGCCGTCGTCCGGCCCTGA
GC
GTAGCAAGAGAAATCCCTCGCTCGGAGTGTCTGTCGGCAGGC-
CGCCCCGAAACCCATATTTA-CAAGGTTGGCCTCGGA
TC
>p19_9
GTTAGCCTTCG-GGCGCAT-CCC-CACCCTCTG-TAACC--GCGGCCCGGATTCGGCG-CCTGGC-
GGGACCACCCCCCG
TTTGCGAGGGGCCCCGCCTGCCGGAATTAGCCAACCCTGCCTGGAAAATTGAAGTCTGAGA
ACAAGTTAAATAAAACAAA
ACTTTCAACAACGGATCTCTTGGTTCTGGCATCGATGAAGAACGCAGCGAAATGCGATAAG
TAATGTGAATTGCAGAATT
CAGTGAATCATCGAATCTTTGAACGCACATTGCGCCCCCTGGTATTCCGGGGGGCACGCCT
GTTCGAGCGCCATTTCTAC

CCTGGAGCCCTGCTCT-
GTGATGGGCCCCGTCCTCGCGGACGGGCCCGAAACCCGTGGGCGCCGTCGTCCGGCCCTGA
GC
GTAGCAAGAGAAATCCCTCGCTCGGAGTGTCTGTCGGCAGGC-
CGCCCCGAAACCCATATTTA-CAAGGTTGGCCTCGGA
TC
>p4_1
GTTAGCCTTCG-GGCGCAT-CCC-CACCCCCTG-TAACC--GCGGCCCGGATTCGGCG-CCTGGC-
GGGACCACCCCCCG
TTTGCGAGGGGCCCCGCCTGCCGGAATTAGCCAACCCTGCCTGGAAAATTGAAGTCTGAGA
ACAAGTTAAATAAAACAAA
ACTTTCAACAACGGATCTCTTGGTTCTGGCATCGATGAAGAACGCAGCGAAATGCGATAAG
TAATGTGAATTGCAGAATT
CAGTGAATCATCGAATCTTTGAACGCACATTGCGCCCCCTGGTATTCCGGGGGGCACGCCT
GTTCGAGCGTCATTTCTAC
CCTGGAGCCCTGCTCT-
GTGATGGGCCCCGTCCTCGCGGACGGGCCCGAAACCCGTGGGCGCCGTCGTCCGGCCCTGA
GC
GTAGCAAGAGAAATCCCTCGCTCGGAGTGTCTGTCGGCAGGC-
CGCCCCGAAACCCATATTTA-CAAGGTTGGCCTCGGA
TC
>p4_2
GTTAGCCTTCG-GGCGCAT-CCC-CACCCCCTG-TAACC--GCGGCCCGGATTCGGCG-CCTGGC-
GGGACCACCCCCCG
TTTGCGAGGGGCCCCGCCTGCCGGAATTAGCCAACCCTGCCTGGAAAATTGAAGTCTGAGA
ACAAGTTAAATAAAACAAA
ACTTTCAACAACGGATCTCTTGGTTCTGGCATCGATGAAGAACGCAGCGAAATGCGATAAG
TAATGTGAATTGCAGAATT
CAGTGAATCATCGAATCTTTGAACGCACATTGCGCCCCCTGGTATTCCGGGGGGCACGCCT
GTTCGAGCGTCATTTCTAC
CCTGGAGCCCTGCTCT-
GTGATGGGCCCCGTCCTCGCGGACGGGCCCGAAACCCGTGGGCGCCGTCGTCCGGCCCTGA
GC
GTAGCAAGAGAAATCCCTCGCTCGGAGTGTCTGTCGGCAGGC-
CGCCCCGAAACCCATATTTA-CAAGGTTGGCCTCGGA
TC
>p28_4
GTTAGCCTTCG-GGCGCAT-CCC-CACCCCCTG-TAACC--GCGGCCCGGATTCGGCG-CCTGGC-
GGGACCACCCCCCG
TTTGCGAGGGGCCCCGCCTGCCGGAATTAGCCAACCCTGCCTGGAAAATTGAAGTCTGAGA
ACAAGTTAAATAAATCAAA
ACTTTCAACAACGGATCTCTTGGTTCTCGCATCGATGAAGAACGCAGCGAAATGCGATAAG
TAATGTGAATTGCAGAATT
CAGTGAATCATCGAATCTTTGAACGCACATTGCGCCCCTTGGTATTCCGGGGGGCATGCCTG
TTCGAGCGTCATTTCTAC
CCTCAAGCCCTGCTTG-
GTGTTGGGCCCCGTCCTCGCGGACGGGCCCGAAACCCGTGGGCGCCGTCGTCCGGCCCTGA
GC
GTAGCAAGAGAAATCCCTCGCTCGGAGTGTCTGTCGGCAGGC-
CGCCCCGAAACCCATATTTA-CAAGGTTGACCTCGGA
TC

>p28_5
GTTAGCCTTCG-GGCGCAT-CCC-CACCCCCTG-TAACC--GCGGCCCGGATTCGGCG-CCTGGC-GGGACCACCCCCCG
TTTGCGAGGGGCCCCGCCTGCCGGAATTAGCCAACCCTGCCTGGAAAATTGAAGTCTGAGAACAAGTTAAATAAATCAAA
ACTTTCAACAACGGATCTCTTGGTTCTCGCATCGATGAAGAACGCAGCGAAATGCGATAAGTAATGTGAATTGCAGAATT
CAGTGAATCATCGAATCTTTGAACGCACATTGCGCCCCTTGGTATTCCGGGGGGCATGCCTGTTCGAGCGTCATTTCTAC
CCTCAAGCCCTGCTTG-GTGTTGGGCCCCGTCCTCGCGGACGGGCCCGAAACCCGTGGGCGCCGTCGTCCGGCCCTGAGC
GTAGCAAGAGAAATCCCTCGCTCGGAGTGTCTGTCGGCAGGC-CGCCCCGAAACCCATATTTA-CAAGGTTGACCTCGGA
TC
>p28_6
GTTAGCCTTCG-GGCGCAT-CCC-CACCCCCTG-TAACC--GCGGCCCGGATTCGGCG-CCTGGC-GGGACCACCCCCCG
TTTGCGAGGGGCCCCGCCTGCCGGAATTAGCCAACCCTGCCTGGAAAATTGAAGTCTGAGAACAAGTTAAATAAATCAAA
ACTTTCAACAACGGATCTCTTGGTTCTCGCATCGATGAAGAACGCAGCGAAATGCGATAAGTAATGTGAATTGCAGAATT
CAGTGAATCATCGAATCTTTGAACGCACATTGCGCCCCTTGGTATTCCGGGGGGCATGCCTGTTCGAGCGTCATTTCTAC
CCTCAAGCCCTGCTTG-GTGTTGGGCCCCGTCCTCGCGGACGGGCCCGAAACCCGTGGGCGCCGTCGTCCGGCCCTGAGC
GTAGCAAGAGAAATCCCTCGCTCGGAGTGTCTGTCGGCAGGC-CGCCCCGAAACCCATATTTA-CAAGGTTGACCTCGGA
TC
>p17_1
GTAAACCTGCA-GGCAGAA-CCC-CAACCCGTG-TAACC--GCGGGCCGGCTTCGGCG-CCTGGC-GGGCCCGCGTCCCG
TTCGCCCGGGGGCCCGCCTCCCGGAGGCTCCAAACCTTGTCTTTTTAATTGAATTCTGAGAACAAGTTAAATAAATCAAA
ACTTTCAGCAACGGATCTCTTGGCTCTCGCATCGATGAAGAACGCAGCGAAATGCGATAAGTAGTGTGAATTGCAGAATT
CAGTGAATCATCGAATCTTTGAACGCACATTGCGCCCGTTGGTATTCCGGAGGGCATGCCTGTCTGAGCGTCATTTCTAC
CCTGGAGCCTTGCTCG-GTGTTGGGCCCTGTCCTCGCGGACTGGCCCGAAACCAGTGGGCGCCGTCGTCTGGCCCTGAGC
GCAGCAAAAGAAACCTCTCGCGCGGACTATCTGTCAGCAGGT-CGCCCTTAAACCCTTTTTT--CAAGGTTGACCTCAGA
TC
>p17_2
GTAAACCTGCA-GGCAGAA-CCC-CAACCCGTG-TAACC--GCGGGCCGGCTTCGGCG-CCTGGC-GGGCCCGCGTCCCG
TTCGCCCGGGGGCCCGCCTCCCGGAGGCTCCAAACCTTGTCTTTTTAATTGAATTCTGAGAACAAGTTAAATAAATCAAA

ACTTTCAGCAACGGATCTCTTGGCTCTCGCATCGATGAAGAACGCAGCGAAATGCGATAAG
TAGTGTGAATTGCAGAATT
CAGTGAATCATCGAATCTTTGAACGCACATTGCGCCCGTTGGTATTCCGGAGGGCATGCCT
GTCTGAGCGTCATTTCTAC
CCTGGAGCCTTGCTCG-
GTGTTGGGCCCTGTCCTCGCGGACTGGCCCGAAACCAGTGGGCGCCGTCGTCTGGCCCTGA
GC
GCAGCAAAAGAAACCTCTCGCGCGGACTATCTGTCAGCAGGT-CGCCCTTAAACCCTTTTTT-
-CAAGGTTGACCTCAGA
TC
>p17_3
GTAAACCTGCA-GGCAGAA-CCC-CAACCCGTG-TAACC--GCGGGCCGGCTTCGGCG-
CCTGGC-GGGCCCGCGTCCCG
TTCGCCCGGGGGCCCGCCTCCCGGAGGCTCCAAACCTTGTCTTTTTAATTGAATTCTGAGAA
CAAGTTAAATAAATCAAA
ACTTTCAGCAACGGATCTCTTGGCTCTCGCATCGATGAAGAACGCAGCGAAATGCGATAAG
TAGTGTGAATTGCAGAATT
CAGTGAATCATCGAATCTTTGAACGCACATTGCGCCCGTTGGTATTCCGGAGGGCATGCCT
GTCTGAGCGTCATTTCTAC
CCTGGAGCCTTGCTCG-
GTGTTGGGCCCTGTCCTCGCGGACTGGCCCGAAACCAGTGGGCGCCGTCGTCTGGCCCTGA
GC
GCAGCAAAAGAAACCTCTCGCGCGGACTATCTGTCAGCAGGT-CGCCCTTAAACCCTTTTTT-
-CAAGGTTGACCTCAGA
TC
>p4_3
GTTAGCCTTCG-GGCGCAT-CCC-CACCCCCTG-TAACC--GCGGCCCGGATTCGGCG-CCTGGC-
GGGACCACCCCCCG
TTTGCGAGGGGCCCCGCCTGCCGGAATTAGCCAACCCTGCCTGGAAAATTGAAGTCTGAGA
ACAAGTTAAATAAAACAAA
ACTTTCAACAACGGATCTCTTGGTTCTGGCATCGATGAAGAACGCAGCGAAATGCGATAAG
TAATGTGAATTGCAGAATT
CAGTGAATCATCGAATCTTTGAACGCACATTGCGCCCCCTGGTATTCCGGGGGGCACGCCT
GTTCGAGCGTCATTTCTAC
CCTGGAGCCCTGCTCT-
GTGATGGGCCCCGTCCTCGCGGACGGGCCCGAAACCCGTGGGCGCCGTCGTCCGGCCCTGA
GC
GTAGCAAGAGAAATCCCTCGCTCGGAGTGTCTGTCGGCAGGC-
CGCCCCGAAACCCATATTTA-CAAGGTTGGCCTCGGA
TC
>p17_4
GTAAGGCTTCG-GGCCCAC-CCC-CAACCCTTG-TAACA--ACTGCCTGGCTTCGGCG-CCTGGC-
GGGACCGCCTCCCG
TTTGCCAGGGGCCCCGCCCGCCGGAGTTAACCAACCTTGTTTTTAAAATTGAATTCTGAGAA
CAAGTTTAACAAAACAAA
ACTTTCAACAACGGATCTCTTGGTTCTCGCATCGATGAAGAACGCAGCGAAATGCGATACC
TAGTGTGAATTGCAGACTT
CAGTGAATCATCGAGTCTTTGAACGCACATTGCGCCCCATGGTATTCCATGGGGCATGCCTG
TCTGAGCGTCGTTTCTAC

CCTCGAGCCAAGCTCA-
GTGTTGGGAACAGGCTATGCGGGCTTTTTCGAAAGACCTGGGCAACGTCGTCCGGACTTGA
GA
GTAGAAATATAAATTTTTAGCACGGACTGTCTTTGGAGAGGC-CGCCCCGAAACCC-
TTTTTAACAAGCTCGACCTCAGA
TC
>p17_5
GTAAGCCTGCG-GGCCCAA-CCC-CAACCCGTG-TAACA--GCGGCCCGGCTTCGGCG-
CCTGGC-GGGCCCGCCTCCCG
TTCGCCAGGGGGCCCGCCCGCCGGAGTTAACCAACCTTGTTTTTAAAATTGAATTCTGAGA
ACAAGTTTAATAAAACAAA
ACTTTCAACAACGGATCTCTTGGTTCTCGCATCGATGAAGAACGCAGCGAAATGCGATACC
TAGTGTGAATTGCAGAATT
CAGTGAATCATCGAGTCTTTGAACGCACATTGCGCCCCATGGTATTCCATGGGGCATGCCTG
TCTGAGCGTCATTTCTAC
CCTCGAGCCAAGCTCA-
GTGTTGGGAACAGGCCATGCGGGCTTGTTCGAAAGACCTGGGCAACGTCGTCCGGACTTGA
GA
GTAGAAATATAAATTTTTAGCACGGACTGTCTTTCGGGAGGC-CGCCCCGAAACCC-
TTTTTAACAAGCTCGACCTCAGA
TC
